# Supplementary material for: Investigating Drug Treatment Costs and Patient Characteristics of Female Breast, Cervical, Colorectal, and Prostate Cancers in Antigua and Barbuda: A Retrospective Data Study (2017–2021)
Source: Int J Environ Res Public Health. 2025 Jun 12;22(6):930. doi: 10.3390/ijerph22060930 (PMC12193607; doi:10.3390/ijerph22060930)
Supplement: Supplementary file 1 [file ijerph-22-00930-s001.zip › Supplementary File S2.pdf]

Supplementary file S2: Results of the performing variable inflation factor (VIF) to assess final models for multicollinearity: VIF of < 10 considered acceptable

Female Breast Cancer		Cervical Cancer	
**Characteristic**	**Variable Inflation Factor**	**Characteristic**	**Variable Inflation Factor**
**Disease stage**		**Age**	
Early Stage	-		1.15
Late Stage	1.11	**Had Diabetes at Presentation**	
**Histological Grade**		No	-
Grade 1	-	Yes	1.32
Grade 2	1.88	**Had Hypertension at Presentation**	
Grade 3	1.72	No	-
Not Stated	1.59	Yes	1.38
**Subtypes**		**Had Cardiovascular Disease at Presentation**	
Luminal A/Luminal B	-	No	-
Triple Negative Breast cancer/HERS/neu Enriched	6.24	Yes	1.46
**Estrogen Receptor Status**			
ER-	-		
ER+	5.98		
**HER2 Status**			
HER2-	-		
HER2+	1.11		

Supplementary file S2. *Cont.*

Colorectal Cancer (n=40)		Prostate Cancer (n=70)	
**Characteristic**	**Variable Inflation Factor**	**Characteristic**	**Variable Inflation Factor**
**Age**		**Age**	
	1.17		1.04
**Disease stage**		**Year**	
Early Stage	-	2017-2019	-
Late Stage	1.28	2020-2021	1.18
**Greatest Dimensions (cm)**		**Family History Status Known**	
≤ 5cm	-	No	-
> 5cm	1.88	Yes	1.14
Not Stated	1.64	**Disease stage**	
**Radiation Therapy Status Known**		Early Stage	-
No	-	Late Stage	1.39
Yes	1.03	**PSA Level (ng/ml)**	
**Had Cardiovascular Disease**		Not Stated	-
No	-	≤20	2.92
Yes	1.16	>20	2.55
**No. of Tracked Payments Made at Hospital**		**Distant Metastases**	
≤ 10	-	Undetermined	-
> 10	1.33	Determined	1.10
