# Supplementary figures and images for: Investigating Drug Treatment Costs and Patient Characteristics of Female Breast, Cervical, Colorectal, and Prostate Cancers in Antigua and Barbuda: A Retrospective Data Study (2017–2021)
Source: Int J Environ Res Public Health. 2025 Jun 12;22(6):930. doi: 10.3390/ijerph22060930 (PMC12193607; doi:10.3390/ijerph22060930)

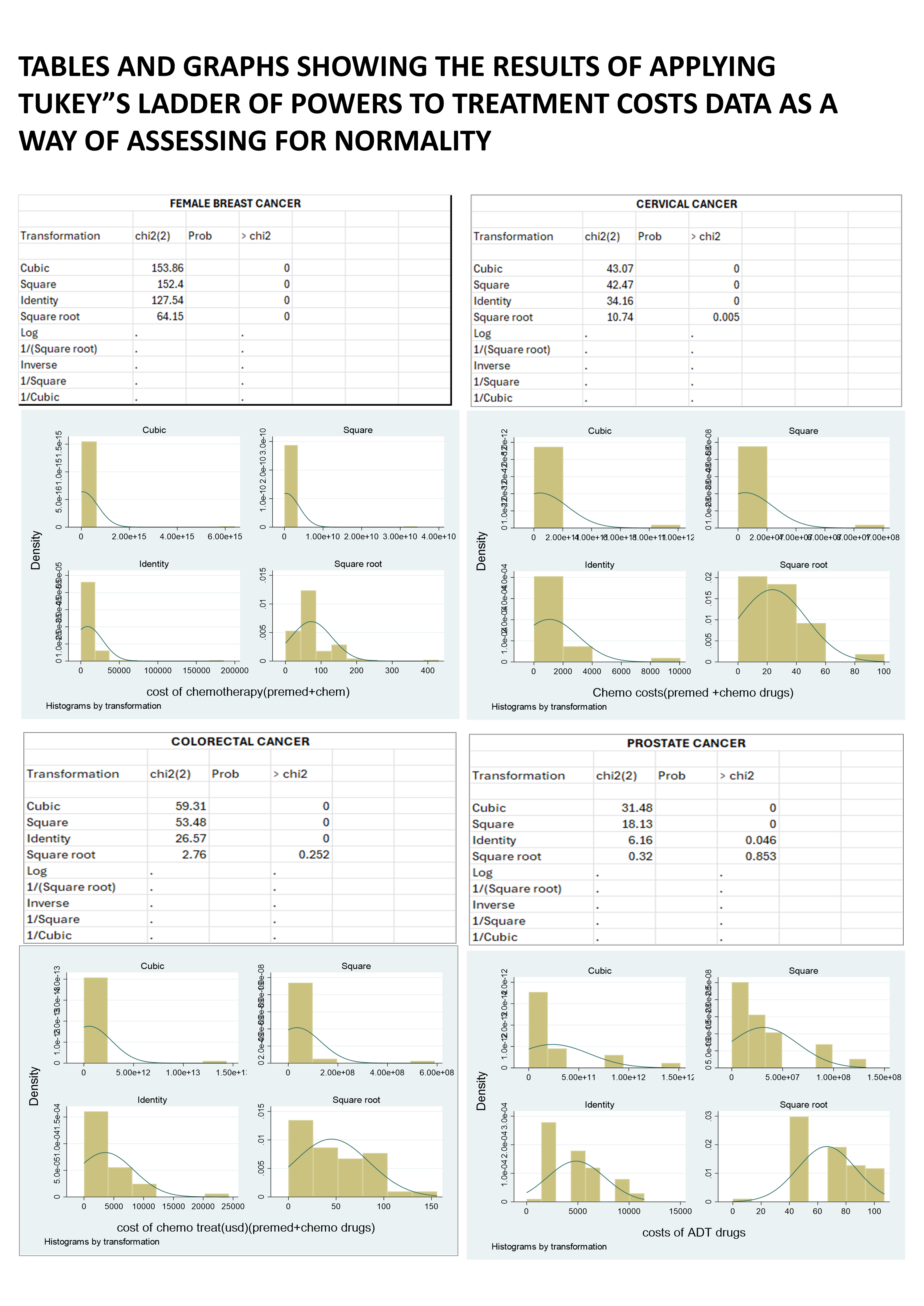

Supplement: Supplementary file 1 [file ijerph-22-00930-s001.zip › Supplementary File S1.tif]

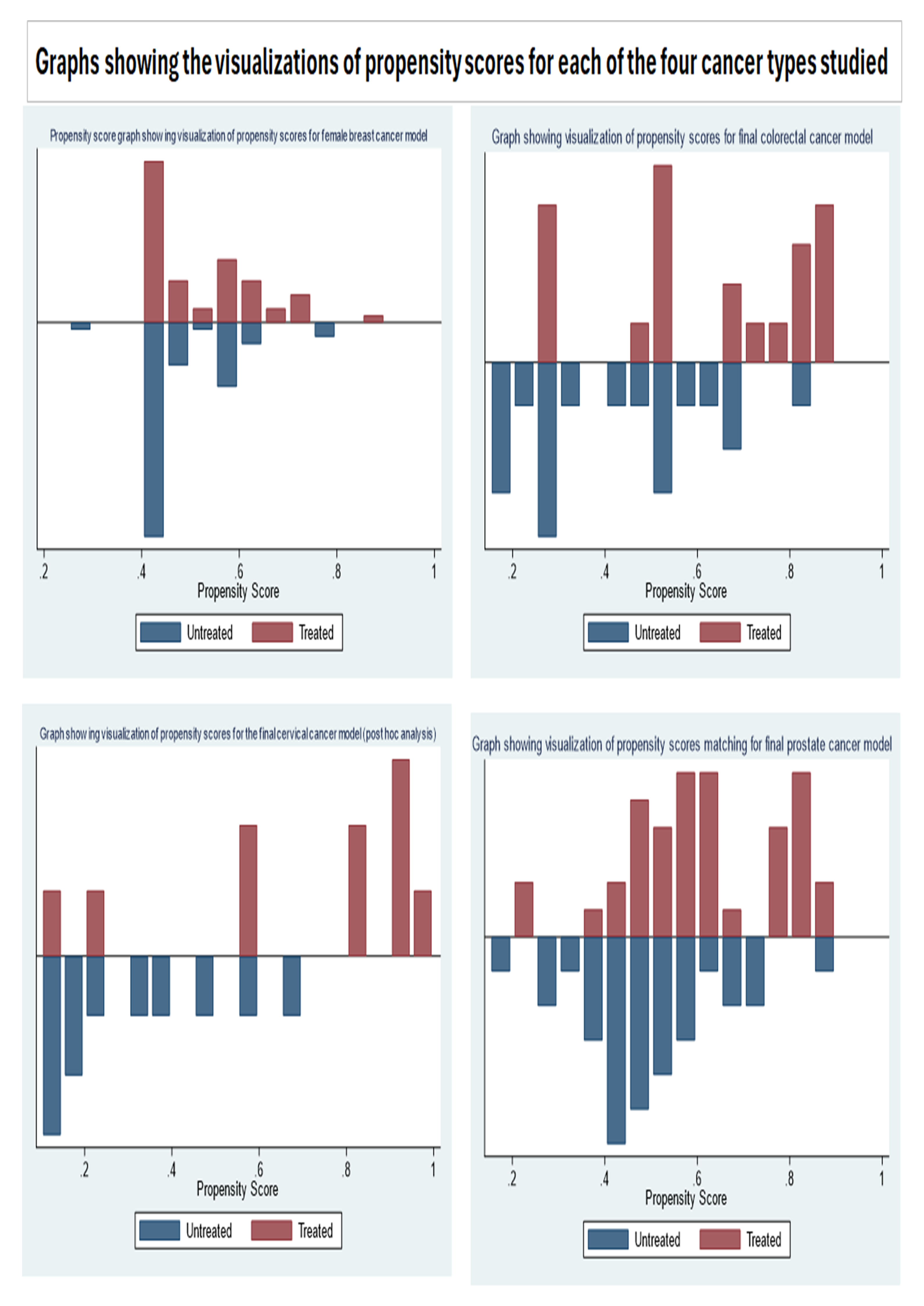

Supplement: Supplementary file 1 [file ijerph-22-00930-s001.zip › Supplementary File S3.tif]
